# Sweet and Sour: The Impact of Differential Glycosylation in Cancer Cells Undergoing Epithelial–Mesenchymal Transition

**DOI:** 10.3389/fonc.2014.00059

**Published:** 2014-03-25

**Authors:** Leonardo Freire-de-Lima

**Affiliations:** ^1^Laboratório de Glicobiologia, Instituto de Biofísica Carlos Chagas Filho, Universidade Federal do Rio de Janeiro, Rio de Janeiro, RJ, Brazil

**Keywords:** epithelial–mesenchymal transition, extracellular matrix, cancer, metastasis, glycosylation, oncofetal fibronectin, glycosyltransferases

## Abstract

Glycosylation changes are a feature of disease states. One clear example is cancer cells, which commonly express glycans at atypical levels or with different structural attributes than those found in normal cells. Epithelial–mesenchymal transition (EMT) was initially recognized as an important step for morphogenesis during embryonic development, and is now shown to be one of the key steps promoting tumor metastasis. Cancer cells undergoing EMT are characterized by significant changes in glycosylation of the extracellular matrix (ECM) components and cell-surface glycoconjugates. Current scientific methodology enables all hallmarks of EMT to be monitored *in vitro* and this experimental model has been extensively used in oncology research during the last 10 years. Several studies have shown that cell-surface carbohydrates attached to proteins through the amino acids, serine, or threonine (*O*-glycans), are involved in tumor progression and metastasis, however, the impact of *O*-glycans on EMT is poorly understood. Recent studies have demonstrated that transforming growth factor-beta (TGF-β), a known EMT inducer, has the ability to promote the up-regulation of a site-specific O-glycosylation in the IIICS domain of human oncofetal fibronectin, a major ECM component expressed by cancer cells and embryonic tissues. Armed with the knowledge that cell-surface glycoconjugates play a major role in the maintenance of cell homeostasis and that EMT is closely associated with glycosylation changes, we may benefit from understanding how unusual glycans can govern the molecular pathways associated with cancer progression. This review initially focuses on some well-known changes found in *O*-glycans expressed by cancer cells, and then discusses how these alterations may modulate the EMT process.

## The Application of Glycobiology in Cancer Research

Cancer is a complex progressive disease that involves a series of genetic–environmental interactions, which cannot occur without the dysfunction of multiple systems, including DNA repair, apoptosis, and immune function. Epigenetic mechanisms responding to numerous internal and external cues in a dynamic ongoing exchange play a key role in mediating environmental influences on gene expression and tumor development. The cancer causing agents (carcinogens) can be present in the air, chemicals, food, water, and even the sunlight people are exposed to. Since epithelial cells line the respiratory and alimentary tracts, cover the skin, and metabolize carcinogens, it is not surprising that over 90% of cancers arise from epithelia (1). The sequencing of the human genome holds benefits for many fields, from molecular medicine to human evolution. The Human Genome Project (HGP) has helped in the understanding of human diseases, including the identification of oncogenes and mutations linked to different forms of cancer (2). However, one major lesson from HGP is that the absolute number of genes encoded by the human genome is not the best parameter for defining its biological complexity. Instead, the complex functions associated with human health and disease are determined by combinatorial expansion of genomic information in the form of post-translational modifications (PTMs).

The quantitative and qualitative control of a vast number of proteins in eukaryotes is maintained by PTMs, such as phosphorylation, ubiquitination, methylation, hydroxylation, proteolysis, N-acetylation, and glycosylation (3, 4). These intricate events require a multitude of specific enzymes that are tissue- and species-specific. The subsequent changes modulate the folding, physicochemical properties, conformation, stability, distribution, immunogenicity, and activity of the proteins (4–6). There is an increasing awareness of post-genome glycosylation as a potentially important form of post-translational protein modification. Glycosylated proteins have long been known to participate in a plethora of biological processes. These include signal transduction, inflammation, viral entry, bacteria–host interactions, cell–cell interactions, and morphogenesis, as well as the development and progression of chronic diseases, such as cancer (7–9). It is well-accepted that in cancer cells, the expression and/or activity of glycosyltransferases is altered, yielding an “aberrant” glycophenotype when compared to their normal counterparts (9, 10). These changes result in structural variations of glycans on glycoproteins and glycolipids, altering their interaction with carbohydrate-binding proteins and modulating the role of the membrane glycan in cell adhesion and signal transduction required for cell motility (11, 12). Furthermore, the aberrant glycosylation of proteins in cancer cells offers an interesting diagnostic perspective in distinguishing what is benign and what is malign (13, 14). Several glycoproteins carrying atypical glycophenotypes have been used as cancer glycobiomarkers (15). Examples include the carcinoembryonic antigen (16), the prostate-specific antigen (17), and the CA-125 antigen (18), used as markers for colorectal, prostate, and ovarian cancers, respectively. The transformation of glycobiology from a descriptive and phenomenological discipline, to one where the regulatory principles are understood and predictably manipulated has opened up new opportunities in the study of cancer and the search for effective therapeutic modalities.

## *O*-Linked Glycan Structures and Their Changes in Cancer Cells

Glycosylation is a main class of PTMs. There are two basic types of protein glycosylation: N-glycosylation and O-glycosylation. These have significant differences in terms of their biosynthesis and structure, as well as their location within the protein chain (7). In *N*-linked glycans, the nitrogen atom in the side chain of asparagine is attached to *N*-acetylglucosamine (GlcNAc). The sequence can be Asn-X-Ser or Asn-X-Thr, where X is any kind of amino acid except proline (19). In *O*-linked glycans, the oxygen atom in the side chain of serine or threonine is attached to *N*-acetylgalactosamine (GalNAc) (20). Furthermore, the glycopeptides Asn-GlcNAc or Ser/Thr-GalNAc may be extended by numerous and specific glycosyltransferases (7).

It is well understood that both *N*- and *O*-linked glycans mediate essential processes in development (21), host–pathogen interactions (22–24), immune cell recognition (25), and cancer development and progression (9, 26). In the case of cancer, alterations in both glycan types frequently modulate the invasive potential of tumor cells and their interactions with stromal partners, including fibroblasts, leukocytes, platelets, and endothelial cells (27). This discussion focuses on the significance of *O*-glycan changes in cancer evolution. However, there are many interesting reviews addressing how atypical *N*-glycans may govern the cancer development and progression [see reviews in Ref. (28, 29)].

**Figure 1:**
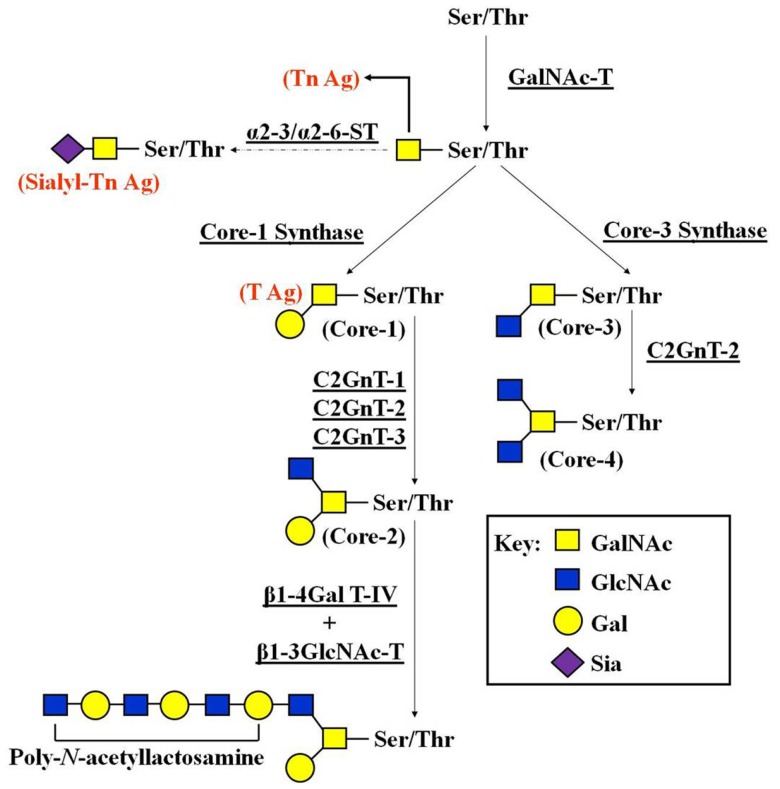
**Pathways of *O*-glycan biosynthesis**. To begin synthesis of core-2 and core-4 *O*-glycans, GalNAc is transferred to a serine or threonine residue in a polypeptide by the GalNAc-T. GalNAc-Ser/Thr is then converted by core-1 synthase to Galβ1-3GalNAc-Ser/Thr (core-1). Core-1 is converted to core-2 by C2GnT-1, -2, and -3. β-1,4-galactosyltransferase IV (β1-4Gal-T IV), together with β-1,3-*N*-acetylglucosaminyltransferase V (β1-3GlcNAc-T V), synthesizes poly-*N*-acetyllactosamine on core-2 *O*-glycans. The number of Galβ1-4GlcNAc disaccharide unit repeats varies depending on the carrier molecules and cell types. GalNAc-Ser/Thr is also converted by β3GnT-6 to core-3. Core-3 is then converted by C2GnT-2 to core-4. As discussed in the main text, the accumulation of truncated versions of expressed oligosaccharides (T-, Tn-, and sialyl Tn-antigens) occurs due to impaired activity of glycosyltransferases involved in the elongation of *O*-linked glycan.

In *O-*linked glycans, the oligosaccharides display considerable structural and antigenic diversity. The GalNAc α-Ser/Thr linkage has been considered a hallmark of mucins, where it occurs in clusters (30). This linkage has also been found in a wide variety of other proteins, such as extracellular matrix (ECM) components (31). The path of O-glycosylation is a key factor in the biological activity of glycoproteins involved with the control of cell differentiation and with the regulation of cell growth through apoptosis and proliferation (32). The addition of GalNAc to Ser or Thr is mediated by glycosyltransferases, known as *N*-acetylgalactosaminyltransferases (GalNAc-Ts) (33). Furthermore, the glycopeptide GalNAc α-Ser/Thr may be extended with additional sugars, including galactose (Gal), GlcNAc, fucose (Fuc), or sialic acid (Sia). There is no glycan extension in cancer cells carrying mutations in the COSMC gene, which encodes for core-1 β3-Gal-T-specific molecular chaperone. Here, the glycopeptide GalNAc α-Ser/Thr, referred to as Tn-antigen, may be sialylated by the action of specific α2-6 sialyltransferases (α2-6 STs), creating the tumor-associated antigen known as sialyl Tn-antigen (34). In general, there are four common *O*-glycan core structures in mammalian tissues (core-1 ⇒ core-4) that depend on the arrangement of the added sugars (Figure 1).

The biosynthesis of core-1 and core-2 structures is entirely dependent on core-1-synthase, a galactosyltransferase (Gal-T), which converts the initial glycopeptide (GalNAcα1-Ser/Thr) to Galβ1-3GalNAcα1-Ser/Thr (core-1 or T-antigen). Core-1 is then converted to core-2 by β1-6 *N*-acetylglucosaminyltransferase (C2GnT). There are three enzymes that can synthesize core-2: C2GnT-1, -2, and -3. Core-2 *O*-glycan is a scaffold for the subsequent production of lactosamine repeats (poly-*N*-acetyllactosamine) on *O*-glycans. Poly-*N*-acetyllactosamine formation is a crucial step for the terminal modification by Sia and Fuc (31). The carbohydrates that contain Sia or Fuc perform important roles in the various steps of tumor formation and tumor progression (35, 36). Enhanced expression of certain Sia types or their linkages is often correlated with the poor prognosis for many human malignancies. Core-3 *O*-glycan is synthesized onto the initial core-1 by core-3 synthase (β3GnT-6), which adds β1-3-linked GlcNAc to GalNAc at the reducing terminus. Core-4 *O*-glycan is then synthesized by the addition of β1-6 GlcNAc to the core-3 acceptor by C2GnT-2 (Figure 1).

Out of the many known types of biosynthetic reactions in *O*-linked glycosylation pathways, only a few structural changes have been frequently connected with tumor development and progression (9, 11, 37–42). Most are either truncated versions of normally expressed oligosaccharides (T-, Tn-, and sialyl Tn-antigens) (Figure 1) or relatively unusual types of outer/terminal oligosaccharide sequences, such as Lewis structures. Such structures arise from the up-regulation of some glycosyltransferases, the down-regulation of competing glycosyltransferases, or the changes in the elongation of the core oligosaccharide structures that create favored acceptors for the capping glycosyltransferases (9, 43). It is well-documented that these truncated antigens show increased expression in tumor cells, which make them potential cancer biomarkers (41), and candidates for the development of vaccines with anti-tumor properties (44). However, little is known about their participation in cancer progression, since few papers have addressed the biological impact of these structures in activating molecular pathways related to cancer cell invasion and metastasis.

It is well described that mucins provide protection on epithelial surfaces, and exert control on cell signaling. Nevertheless, mucins in cancer cells carrying aberrant *O*-glycophenotypes induce profound tumor-promoting effects, partly mediated through interactions with their glycan moieties (45). MUC1 is greatly glycosylated in benign cells of the breast, prostate, ovary, and pancreas, and is minimally expressed and limited to the apical side of the glands. Transformation of these cells into malignant cells is associated with the over-expression of MUC1, and dysregulation of *O*-glycans of MUC1 (46). Previous studies showed that the binding of galectin-3 to MUC1 bearing the oncofetal Thomsen–Friedenreich antigen [Galβ1,3 GalNAc-α (TF)] increases the cancer cell adhesion to the endothelium (47). In addition, recent reports demonstrated that the VNTR sequence found in the extracellular domain of MUC1 may carry altered *O*-glycophenotypes, which could be responsible to modulate cancer cell invasion and migration (48). Recently, Wang and colleagues (49) described the strong pro-carcinogenic potential of the *N*-acetylgalactosaminyltransferase-3 (GalNAc-T3) in advanced epithelial ovarian cancer (EOC). The authors demonstrated that the knockdown of GalNAc-T3, a possible GalNAc-T involved in oncofetal fibronectin (onfFN) biosynthesis (50), promoted an increase of the cell adhesion molecules β-catenin and E-cadherin. These are normally suppressed by MUC1 in aggressive cancer cells, thus supporting the role of the GalNAc-T3/MUC1 axis in EOC invasion (49). Although several papers have connected the altered expression of MUC1 to epithelial–mesenchymal transition (EMT) (51–54), the impact of atypical *O*-glycans carried by MUC1 on cancer cells undergoing EMT is still unknown.

Another example is the sialomucin CD43, which is one of the most prevalent *O*-glycosylated mucins expressed by leukocytes. It was found to be expressed by human adenomas and carcinomas, but not in normal epithelial cells (55). In addition, Fu and colleagues (56) recently demonstrated that human lung carcinoma cells became more sensitive to TNF-α-induced cell death after CD43 knockdown. However, the impact of CD43 on EMT is unrecognized. So far, the roles of *O*-linked glycans in promoting tumor metastasis have been investigated by focusing on selectin-mediated interactions between tumor cells and other cells types (57–60). Sialyl Lewis antigens, which may be found in core-2 *O*-glycans, bind to selectins expressed on endothelial cells, favoring the extravasation of cancer cells into metastasis target organs (61, 62). Recently (63, 64), intracellular glycoproteins modified by a single *O*-linked-beta-*N*-acetylglucosamine (*O*-GlcNAc) have shown to modulate cell–cell adhesion and cell motility. The involvement of these *O*-GlcNAcylated glycoproteins on EMT process has been described (65).

Since altered glycoconjugates expressed by cancer cells are typically detected during fetal development, they are known as oncofetal antigens. These proteins are often measurable in the blood and may be used for cancer diagnosis and to monitor the effectiveness of cancer treatment. Examples include α-fetoprotein, which is produced by hepatocellular carcinoma and some germ cell tumors (66), the carcinoembryonic antigen, which is elevated in people with colon cancer (67), and *O*-glycosylated onfFN, which was discovered in 1985 by Matsuura and Hakomori (68).

## *O*-Glycosylated Oncofetal Fibronectin: A Major ECM Component Expressed by Cancer Cells and Embryonic Tissues

**Figure 2:**
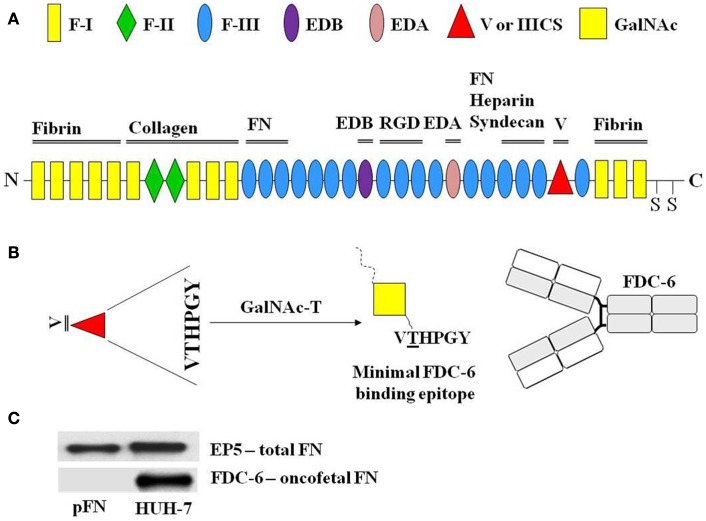
**The modular structure of fibronectin and its binding domains**. FN consists of type I, type II, and type III repeats. Sets of repeats constitute binding domains for fibrin, FN, syndecan, collagen, and heparin, as indicated. SS indicates the C-terminal cysteines that form the dimer. The EDB and EDA domains that are produced by alternative splicing are indicated **(A)**. Between type I and type III repeats there is another alternatively spliced segment, which is termed IIICS or variable region. During the alternative splicing, five different IIICS variants may be generated (V0, V64, V89, V95, and V120). Excluding V0, all others variants may hold the hexapeptide (VTHPGY) required to create the *O*-glycosylated onfFN, which is produced from the addition of a GalNAc unit at the Thr residue of the hexapeptide (V*T*HPGY) by a specific GalNAc-T. It creates the oncofetal epitope required for FDC-6 binding **(B)**. As described, FDC-6 reacts exclusively with the *O*-glycosylated onfFN, but not with non-*O*-glycosylated FN or plasma FN (pFN) **(C)**.

Since the same cell behaviors and processes that are indispensable for embryonic development are also essential for cancer progression (69), it is not surprising that in the last two decades, we have seen that cancer biology and development biology “walk hand in hand” (70, 71). The notion that the local microenvironment plays an essential role in regulating cell behavior, a central theme in classical embryology, has become increasingly accepted in the cancer biology field (72–74). Previous studies have been dedicated to determining how cellular components of the niche initiate and promote cancer development (75). However, more recent progress has also highlighted the importance of non-cellular components of the niche, especially the ECM during cancer progression (76–79). The ECM is a mixture of many different molecular components that vary between organisms, tissues within one organism, and sometimes with the developmental age. It is not a static structure, but is instead being constantly remodeled by the cells within it and around it, by the opposing influences of ECM synthesis and destruction by proteolytic enzymes. The ECM is composed of three main classes of macromolecules: structural proteins (collagen and elastin), specialized proteins (fibrillin, laminin, and FN), and glycosaminoglycans. Amongst all of the ECM components in vertebrates, FN has received considerable attention for its role in cell–matrix interactions. This adhesive glycoprotein is critically important in vertebrate development, since gene disruption studies demonstrated early embryonic lethality in mice without the FN gene. Although FN has been studied for more than two decades, this remarkably complex molecule is still the subject of exciting discoveries. FN usually exists as a dimer, composed of two nearly identical ~250 kDa subunits linked covalently together near their C-termini by a pair of disulfide bonds. FN binds different ECM components and mediates homotypic interactions (Figure 2A). Until the mid-80s, two major types of FN were known: the plasma (pFN) and cellular FN (cFN), which was found in the pericellular matrix or secreted in the culture medium of fibroblasts. In 1985, Matsuura and Hakomori developed a monoclonal antibody (mAb) termed FDC-6, which was initially reactive with cFN expressed by fibroblasts, but not with pFN. Furthermore, the authors demonstrated that FNs isolated from normal adult tissues were not reactive with FDC-6, whereas FNs isolated from established cell lines, such as fetal and cancer tissues carried an extra structure, which favored FDC-6 binding. This FN became known as onfFN. Proteolytic digestion of glycoproteins showed that the structure defined by FDC-6 antibody was present in the variable (V) region or IIICS domain of FN (68). After characterization of the primary structure of V region, Matsuura and colleagues isolated the specific structure for FDC-6 binding. The comparison of this structure with other structures that lack reactivity with FDC-6 showed that the FDC-6 binding epitope was composed of a GalNAc linked to Thr in the hexapeptide VTHPGY. In addition, the authors demonstrated that the reactivity is dependent on the combination of both the sugar moiety and the peptide unit, since neither the sugar nor the peptide moiety alone could form the epitope structure (80). Further studies showed that the FDC-6 non-reactive synthetic peptide containing the VTHPGY sequence can be converted into FDC-6-reactive form. This happens after the incubation with GalNac-T and UDP-[^3^H]GalNAc in the homogenate of carcinoma cell lines and the human fetal fibroblast cell lines. Such a conversion did not take place when the same enzyme fraction of normal adult tissue was incubated with the VTHPGY peptide under the same conditions. Thus, the occurrence of GalNAc-T recognizing the VTHPGY peptide sequence was shown to be specific for fetal and cancer tissues, and absent in normal adult tissues (81).

It is now known that FN isoforms are developmentally regulated and generated as a result of alternative splicing of the primary FN transcript (82). The mature FN molecules comprise of a series of repeating amino acid sequences, termed as F-I, F-II, and F-III structural modules. Twelve F-I modules make up the amino-terminal and carboxy-terminal region of the molecule, and are involved mainly in fibrin and collagen binding. Only two F-II modules are found in FN. They are instrumental in binding collagen. The most abundant module in FN is F-III, which contains the RGD FN receptor recognition sequence along with binding sites for other integrins and ECM components (Figure 2A) (71). Depending on the tissue type and/or cellular conditions, the FN molecule is made up of 15-17 F-III modules. The V region, which does not fall into any of these categories, along with extra domain B (EDB) and extra domain A (EDA) (both F-III modules), is regulated through alternative splicing of FN pre-mRNA (71). The V region is located between F-I and F-III modules and can generate five (V0, V64, V89, V95, and V120) different variants after the alternative splicing (71, 72). All variants, except V0 may contain the hexapeptide (VTHPGY) that can be *O*-glycosylated on its Thr residue by a specific GalNAc-T, creating the oncofetal epitope required for the FDC-6 binding (Figure 2B).

As described above, FDC-6 reacts exclusively with *O*-glycosylated onfFN, but not with non-*O*-glycosylated FN or plasma FN (pFN) (Figure 2C). onfFN has been identified within several human tumors and developmental tissues (83–85). Furthermore, a few recent reports have demonstrated that both *O*-glycosylated and non-*O*-glycosylated FNs display distinct functionalities in cancer cells undergoing EMT (see section below) (86–88). However, further studies are necessary to clarify their divergent roles in cancer evolution.

Although the development of a cancer cell arises from epigenetic alterations, as well as somatic mutations in essential genes for cell physiology, the selective pressures that take place in the tumor microenvironment are crucial for the emergence of transformed cells with different genotypes and/or glycophenotypes. These events can modulate signaling pathways that exert control on proliferation, motility, and half-life, which create conditions for such cells to acquire invasive properties. For a long time, little was known about the biology of cancer cells with metastatic properties. However, with the advent of experimental models, which allowed the studies of molecular pathways triggered in invasive cancer cells has generated valuable information that can be used for future diagnostic and therapeutic purposes.

## The Role of EMT in Cancer Progression and Metastasis

**Figure 3:**
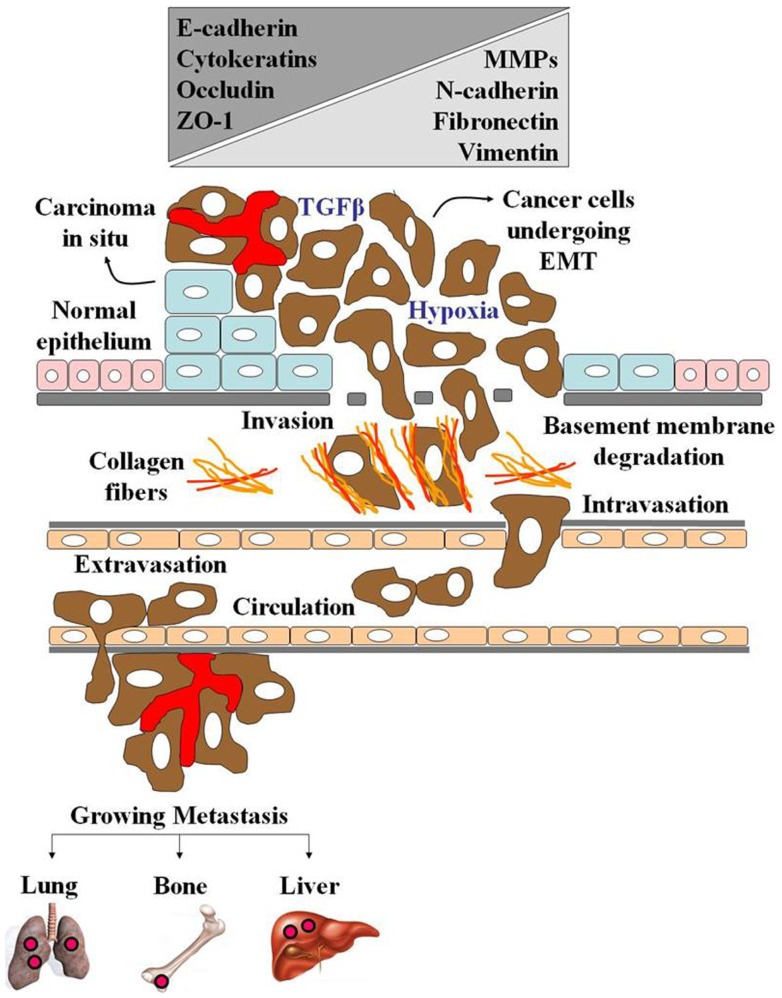
**The epithelial–mesenchymal transition**. EMT is a morphogenetic process in which epithelial cells lose their characteristics and gain mesenchymal properties during embryogenesis and during progression of cancer. Carcinoma cells acquire a mesenchymal-like state in order to facilitate their migration and invasion. The EMT process is induced and regulated by effectors such as growth factors (TGFβ), as well by hypoxia. It is characterized by loss of epithelial markers (E-cadherin, cytokeratins, occludin, and ZO-1), gain of mesenchymal markers (N-cadherin, vimentin, and fibronectin) and high expression of MMPs, which help cancer cells grow and spread.

Given the ultimate diversity of tissues in a mature organism, cells can adopt different fates. This process, known as differentiation, provides cells with their distinct identities and specialized functions. Initially, it was thought that differentiation was a one-way process, and those terminally differentiated cells, i.e., cells of the epithelium, were unable to lose their individuality and differentiate toward a cell type of another lineage. However, more recent studies have shown that cells of the epithelium can dedifferentiate toward a mesenchymal state, enabling the cells to migrate, and form new structures at a distant site. This phenomenon termed EMT is a crucial biological event that takes place during embryonic development and wound healing. Next to these physiological events, EMT pathways are also activated during diseases. Cancer cells can go through EMT to detach from the primary tumor, migrate toward the vasculature, get transported by the blood circulation through the body, and settle at a distant site where metastatic foci can be formed (89–92). Nowadays, all hallmarks of EMT can be monitored *in vitro*, and this unique experimental model has been used to shed light on the molecular, biochemical, and biomechanical cues of invasive cells. In literature, three different subtypes of EMT are recognized. Although EMT, by definition, results in the formation of migratory mesenchymal cells, each subtype represents a specific biological event (93). The earliest EMT (type 1 EMT) is highly regulated and is associated with embryonic implantation and organ formation. Type 2 EMT is associated with inflammation and fibrosis, and is now increasingly recognized in adult pathological conditions. In the context of fibrosis, the inflammation persists and fibroblastic cells accumulate in the tissue, secreting large concentrations of ECM components. The deposition of these ECM components inhibits organ function and can ultimately lead to organ failure and organ destruction (94). Type 3 EMT is associated with cancer progression and occurs in epithelial tumors (95, 96). Figure 3 illustrates how the type 3 EMT may modulate the cancer cell progression.

In normal epithelial tissues, the cells are shown tightly juxtaposed and form a polarized sheet with differing apical and basal surfaces. When the genes involved in the control of cell growth and cell death undergo epigenetic alterations and somatic mutations, the mutated cells proliferate in an uncontrolled way, and favor the development of carcinoma *in situ* (Figure 3). Growth factors, such as TGF-β, and the compromised oxygen demand (hypoxia condition) in the tumor microenvironment activates the molecular pathways related to EMT. During this process, EMT master regulators, such as the transcription factors Twist, Snail, and SIP1, lead to dramatic changes in gene expression profiles and cellular behavior. Twist, Snail, and SIP1 repress E-cadherin expression via E-boxes in its promoter and trigger expression of an entire EMT transcriptional program. The cells show a spindle-like-shape, as well a significant reduction of epithelial markers (E-cadherin, cytokeratins, occludin, and ZO-1), which is accompanied by the emergence of mesenchymal markers (N-cadherin, vimentin, and FN). In addition, the transformed cells secrete metalloproteinases (MMPs), which help degrade the basal membrane components and also expresses high levels of mesenchymal integrins, which may facilitate their interaction with ECM components, such as collagen fibers (Figure 3). Finally, the transformed cells are able to reach the bloodstream and colonize distant organs and tissues inducing metastasis (Figure 3). Over 45 years, it is well-accepted that atypical glycosylation may be detected in all types of experimental and human cancers. However, although many tumor-associated antigens have been used as tumor glycobiomarkers, their role on cancer evolution is not well studied. In part, this can be explained by the difficulties in closely following the biochemical and molecular events of tumor development and progression *in vivo*. Along this line, the EMT process has helped us to unveil the cues of the altered glycosylation in invasive cancer cells.

## EMT: The Glycan Connection

There is universal agreement that most of the early events in the emergence of neoplasia involve genetic alterations, and it seems unlikely that altered glycosylation *per se* plays a major role at this stage. However, few reports have demonstrated how these atypical glycosylations may govern the molecular pathways related to cancer cell invasion and metastasis. The impact of altered glycans modulating cancer cell behavior is of central significance in recent cancer research. However, little attention has been given to this topic, mainly because the structural and functional concepts of glycosylation in cancer are more complex than those for certain proteins and their genes in defining cancer cell phenotypes. Pioneering papers published by Hakomori’s group demonstrated that the EMT process may be used to shed light on the impact of atypical glycans expressed by invasive epithelial cells (86, 87, 97–99). After the first paper published in 2009, where the participation of specific glycosphingolipids (GSLs) in human and mouse mammary epithelial cells undergoing EMT was demonstrated, a few papers discussing the impact of atypical glycans, as well as the altered expression of glyco-genes have been published (100–103). However, the implication of the unusual *O*-linked glycans on EMT process has not been well explored. So far, the impact of altered *O*-glycans on EMT has been addressed to the *O*-glycosylated onfFN. Although onfFN was discovered almost 30 years ago (68), until very recently, there had been no studies that describe its role in cancer biology. Over the last few years, the *O*-glycosylated onfFN has only been used for diagnostic purposes (104).

Recent studies brought to light the involvement of onfFN during the EMT process. The first report demonstrated the up-regulation of onfFN in human prostate epithelial cells treated with TGF-β. The authors observed that the treated cells expressed high levels of mRNA for FN carrying the IIICS domain, which contains the VTHPGY sequence (86). Although it is recognized that the incubation of the hexapeptide cited above with the microsomal fraction of cancer cells favored the appearance of the oncofetal epitope, the identity of the GalNAc-T responsible for this phenomenon remained unknown for a long time. Previous biochemical studies revealed that both GalNAc-T3 and/or GalNAc-T6 recombinant enzymes were able to glycosylate the VTHPGY sequence, suggesting that one or both glycosyltransferases would participate in onfFN biosynthesis (50). To analyze the relevance of GalNAc-T3 and/or GalNAc-T6 on EMT, Freire-de-Lima and colleagues (86) silenced the expression of both glycosyltransferases and observed that the double knockdown cells failed to undergo TGF-β-induced EMT. These were the first results that evinced a cell system where both GalNAc-T3 and GalNAc-T6 were involved in onfFN biosynthesis, as well as indirectly demonstrating the importance of *O*-glycosylated onfFN in human epithelial cells undergoing EMT. To check the main type of FN expressed during the EMT process, a new hybridoma, YKH1, which produces a mAb directed against the non-*O*-glycosylated VTHPGY sequence found in FNs expressed by normal tissues (norFN) has been produced (87). The comparative results revealed that TGF-β-treated cells expressed a significant amount of onfFN when compared with non-treated cells. On the other hand, there was no observable change on YKH1 binding between the control and TGF-β treated cells, suggesting that the majority, if not all of the FN produced during EMT is onfFN (87). Additionally, both norFN and onfFN were purified by immunoaffinity columns to directly evaluate their relevance on EMT. Interestingly, the authors observed that only the *O*-glycosylated onfFN was able to induce the EMT-related events (87). Although the major ligands for onfFN, as well as the molecular mechanisms triggered by this unusual glycoprotein are still unknown, the results described above have a significant biological impact, suggesting that onfFN may be further used as a possible candidate for the development of vaccines with anti-carcinogenic properties.

Increasing evidence suggests that a metabolic disturbance, such as hyperglycemia, is closely linked to cancer development and progression. Several epidemiological studies have confirmed that diabetic patients are more susceptible to cancer development when compared with healthy individuals (105–108). Furthermore, diabetic patients have a poorer prognosis than those non-diabetic patients with the same types of cancer (109, 110). The exact mechanism for the increased cancer risk in patients with diabetes is unknown. Due to a potential correlation between diabetes and cancer, studies evaluating the cancer risk of medications used to treat diabetes are emerging (111, 112). However, given the importance of glycoconjugates on cell biology, the possibility that atypical glycans in diabetic individuals may support the tumor development and/or evolution cannot be excluded. The up-regulation of glucose transporters (Gluts), which contributes to increased influx of glucose, is one of the metabolic facets adopted by cancer cells. It is believed that ~95% of glucose is deviated to the glycolytic pathway, and ~3–5% is skewed to the hexosamine biosynthetic pathway (HBP) that generates the activated monosaccharides UDP-GlcNac and UDP-GalNAc, which are essential for the biosynthesis of *N*- and *O*-linked glycans respectively. Following this thought process; it would not be surprising if cancer cells maintained in a high glucose condition were able to express more *O*-glycosylated onfFN than those cells kept in a normoglycemic condition.

Over the last few years, several papers evinced that the maintenance of different cell types in high glucose condition promoted the up-regulation of FN expression (113, 114). Furthermore, recent works have demonstrated that a high glucose condition contributes to the increased production of TGF-β *in vitro* by diverse cell lines (115–117). It is well described that FN is an important mesenchymal marker, and that TGF-β is a potently known EMT inducer. However, no studies have shown a direct link between a hyperglycemic condition and the EMT process. Furthermore, it is not known what type of FN would be expressed in a hyperglycemic condition. We recently demonstrated that the maintenance of a human adenocarcinoma cell line (A549) in hyperglycemic, but not in normoglycemic condition, was able to induce the EMT process, which was accompanied by the up-regulation of the mRNA for FN transcripts carrying the IIICS domain and onfFN expression (88). In addition, we have shown that these phenomena seem to be fully dependent of the endogenous TGF-β produced in high glucose, since the neutralization of TGF-β by anti-TGF-β antibodies abrogated the onfFN expression. These results have important implications, since they make a direct link between diabetes, a metabolic disorder which affects millions of people worldwide, and carcinogenesis. It also reinforces the idea that aberrant glycosylation of glycoconjugates in diabetic patients may have a key role in oncogenesis and tumor progression. Further studies are going on in our lab to clarify this hypothesis.

## Conclusion

This review has summarized how unusual *O*-glycans may influence cancer cell behavior, focusing on the effects mediated by the *O*-glycosylated onfFN in EMT, a biological phenomenon that has revolutionized the study of carcinogenesis. As commented above, several papers have described altered *O*-glycophenotypes adopted by cancer cells. However, there are few reports regarding how these alterations may govern the molecular pathways related to the cancer evolution. The involvement of atypical glycans in cancer cells undergoing EMT is a topic still not well studied, but the area is very promising, given the relevance of glycoconjugates in the maintenance of cell homeostasis, as well by the fact that EMT precedes cell metastasis. Research efforts over the last few years have concentrated on the impact of an unusual *O*-linked glycan in the FN structure. Future results will be necessary to support previous observations, but it is plausible to speculate that onfFN may be further used as a promising candidate for an anti-cancer vaccine. Basic Local Alignment Search Tool (BLAST) analysis (118) revealed that human kallikrein 12 (h-klk12), a serine protease expressed by adult and fetal tissues (119), share the same VTHPGY sequence that is *O*-glycosylated in the IIICS domain of human onfFN. It has been well described that h-klk12 modulates many physiological processes (120, 121), as well tumor cell invasion and metastasis (122, 123). Previous studies have shown that cancer cells express kallikreins with atypical glycophenotypes (124). Knowing this, it is reasonable to speculate that h-klk12 may act as substrate for GalNAc-T3 and/or GalNAc-T6 to create the oncofetal epitope recognized by FDC-6. However, this has never been demonstrated. In addition to FN, other glycoproteins associated with cell adhesion, such as cytokeratins (125, 126), plakoglobin (127, 128), E-cadherin (129), and beta-catenin (63, 130) are known to express their peculiar *O*-glycophenotypes. Although the EMT process is deeply associated with glycosylation changes, there is little information regarding the impact of the atypical *O*-linked glycans adopted by these glycoproteins in cancer cells undergoing EMT. Given the importance of these *O*-glycophenotypes in the maintenance of epithelial cell polarity, it would be interesting to further explore this topic in cancer biology. Besides reinforcing the idea that oncoglycobiology is an important biological field, one of the major goals of this review is to show that the EMT model may render useful information about the glycosylation changes in invasive and metastatic cancer cells, which may be additionally used for therapeutic and diagnostic purposes.

## Conflict of Interest Statement

The author declares that the research was conducted in the absence of any commercial or financial relationships that could be construed as a potential conflict of interest.

## References

[1] Birchmeier W Behrens J Weidner KM Hulsken J Birchmeier C Epithelial differentiation and the control of metastasis in carcinomas. Curr Top Microbiol Immunol (1996) 213(Pt 2):117–35 9053287 10.1007/978-3-642-61109-4_6

[2] Alfoldi J Lindblad-Toh K Comparative genomics as a tool to understand evolution and disease. Genome Res (2013) 23(7):1063–8 10.1101/gr.157503.113 23817047 PMC3698499

[3] Karve TM Cheema AK Small changes huge impact: the role of protein posttranslational modifications in cellular homeostasis and disease. J Amino Acids (2011) 2011:207691 10.4061/2011/207691 22312457 PMC3268018

[4] Seo J Lee KJ Post-translational modifications and their biological functions: proteomic analysis and systematic approaches. J Biochem Mol Biol (2004) 37(1):35–44 10.5483/BMBRep.2004.37.1.035 14761301

[5] Woodsmith J Kamburov A Stelzl U Dual coordination of post translational modifications in human protein networks. PLoS Comput Biol (2013) 9(3):e1002933 10.1371/journal.pcbi.1002933 23505349 PMC3591266

[6] Schuldt A Post-translational modification: a SUMO protease for stress protection. Nat Rev Mol Cell Biol (2013) 14(5):263 10.1038/nrm3569 23594955

[7] Spiro RG Protein glycosylation: nature, distribution, enzymatic formation, and disease implications of glycopeptide bonds. Glycobiology (2002) 12(4):43R–56R 10.1093/glycob/12.4.43R 12042244

[8] Hakomori S Aberrant glycosylation in cancer cell membranes as focused on glycolipids: overview and perspectives. Cancer Res (1985) 45:2405–14 3886132

[9] Hakomori S Glycosylation defining cancer malignancy: new wine in an old bottle. Proc Natl Acad Sci U S A (2002) 99(16):10231–3 10.1073/pnas.172380699 12149519 PMC124893

[10] Kim YJ Varki A Perspectives on the significance of altered glycosylation of glycoproteins in cancer. Glycoconj J (1997) 14(5):569–76 10.1023/A:1018580324971 9298689

[11] Ono M Hakomori S Glycosylation defining cancer cell motility and invasiveness. Glycoconj J (2004) 20(1):71–8 10.1023/B:GLYC.0000018019.22070.7d 14993838

[12] Park S-Y Yoon S-J Freire-de-Lima L Kim J-H Hakomori S Control of cell motility by interaction of gangliosides, tetraspanins, and epidermal growth factor receptor in A431 vs. KB epidermoid tumor cells. Carbohydr Res (2009) 344(12):1479–86 10.1016/j.carres.2009.04.032 19559406

[13] Peracaula R Barrabes S Sarrats A Rudd PM de Llorens R Altered glycosylation in tumours focused to cancer diagnosis. Dis Markers (2008) 25(4–5):207–18 10.1155/2008/797629 19126965 PMC3827805

[14] Meany DL Chan DW Aberrant glycosylation associated with enzymes as cancer biomarkers. Clin Proteomics (2011) 8(1):7 10.1186/1559-0275-8-7 21906357 PMC3170274

[15] Drake PM Cho W Li B Prakobphol A Johansen E Anderson NL Sweetening the pot: adding glycosylation to the biomarker discovery equation. Clin Chem (2010) 56(2):223–36 10.1373/clinchem.2009.136333 19959616 PMC2849286

[16] Duffy MJ Carcinoembryonic antigen as a marker for colorectal cancer: is it clinically useful? Clin Chem (2001) 47(4):624–30 11274010

[17] Peracaula R Tabares G Royle L Harvey DJ Dwek RA Rudd PM Altered glycosylation pattern allows the distinction between prostate-specific antigen (PSA) from normal and tumor origins. Glycobiology (2003) 13(6):457–70 10.1093/glycob/cwg041 12626390

[18] Saldova R Struwe WB Wynne K Elia G Duffy MJ Rudd PM Exploring the glycosylation of serum CA125. Int J Mol Sci (2013) 14(8):15636–54 10.3390/ijms140815636 23896595 PMC3759877

[19] Weerapana E Imperiali B Asparagine-linked protein glycosylation: from eukaryotic to prokaryotic systems. Glycobiology (2006) 16(6):91R–101R 10.1093/glycob/cwj099 16510493

[20] Kleene R Schachner M Glycans and neural cell interactions. Nat Rev Neurosci (2004) 5(3):195–208 10.1038/nrn1349 14976519

[21] Ohtsubo K Marth JD Glycosylation in cellular mechanisms of health and disease. Cell (2006) 126(5):855–67 16959566 10.1016/j.cell.2006.08.019

[22] Freire-de-Lima L Oliveira IA Neves JL Penha LL Alisson-Silva F Dias WB Sialic acid: a sweet swing between mammalian host and *Trypanosoma cruzi*. Front Immunol (2012) 3:356 10.3389/fimmu.2012.00356 23230438 PMC3515882

[23] Szymanski CM Wren BW Protein glycosylation in bacterial mucosal pathogens. Nat Rev Microbiol (2005) 3(3):225–37 10.1038/nrmicro1100 15738950

[24] Freire-de-Lima L Alisson-Silva F Carvalho ST Takiya CM Rodrigues MM DosReis GA *Trypanosoma cruzi* subverts host cell sialylation and may compromise antigen-specific CD8+ T cell responses. J Biol Chem (2010) 285(18):13388–96 10.1074/jbc.M109.096305 20106975 PMC2859498

[25] Marth JD Grewal PK Mammalian glycosylation in immunity. Nat Rev Immunol (2008) 8(11):874–87 10.1038/nri2417 18846099 PMC2768770

[26] Dube DH Bertozzi CR Glycans in cancer and inflammation – potential for therapeutics and diagnostics. Nat Rev Drug Discov (2005) 4(6):477–88 10.1038/nrd1751 15931257

[27] Mueller MM Fusenig NE Friends or foes – bipolar effects of the tumour stroma in cancer. Nat Rev Cancer (2004) 4(11):839–49 10.1038/nrc1477 15516957

[28] Lau KS Dennis JW N-glycans in cancer progression. Glycobiology (2008) 18(10):750–60 10.1093/glycob/cwn071 18701722

[29] Taniguchi N Korekane H Branched N-glycans and their implications for cell adhesion, signaling and clinical applications for cancer biomarkers and in therapeutics. BMB Rep (2011) 44(12):772–81 10.5483/BMBRep.2011.44.12.772 22189679

[30] Tian E Ten Hagen KG Recent insights into the biological roles of mucin-type O-glycosylation. Glycoconj J (2009) 26(3):325–34 10.1007/s10719-008-9162-4 18695988 PMC2656418

[31] Tsuboi S Hatakeyama S Ohyama C Fukuda M Two opposing roles of O-glycans in tumor metastasis. Trends Mol Med (2012) 18(4):224–32 10.1016/j.molmed.2012.02.001 22425488 PMC3356160

[32] Patsos G Andre S Roeckel N Gromes R Gebert J Kopitz J Compensation of loss of protein function in microsatellite-unstable colon cancer cells (HCT116): a gene-dependent effect on the cell surface glycan profile. Glycobiology (2009) 19(7):726–34 10.1093/glycob/cwp040 19293232

[33] Gill DJ Clausen H Bard F Location, location, location: new insights into O-GalNAc protein glycosylation. Trends Cell Biol (2011) 21(3):149–58 10.1016/j.tcb.2010.11.004 21145746

[34] Ju T Wang Y Aryal RP Lehoux SD Ding X Kudelka MR Tn and sialyl-Tn antigens, aberrant O-glycomics as human disease markers. Proteomics Clin Appl (2013) 7(9–10):618–31 10.1002/prca.201300024 23857728 PMC5808880

[35] Fernandez-Rodriguez J Feijoo-Carnero C Merino-Trigo A Paez de la Cadena M Rodriguez-Berrocal FJ de Carlos A Immunohistochemical analysis of sialic acid and fucose composition in human colorectal adenocarcinoma. Tumour Biol (2000) 21(3):153–64 10.1159/000030122 10754466

[36] Bathi RJ Nandimath K Kannan N Shetty P Evaluation of glycoproteins as prognosticators in head and neck malignancy. Indian J Dent Res (2001) 12(2):93–9 11665403

[37] Dennis JW Laferte S Tumor cell surface carbohydrate and the metastatic phenotype. Cancer Metastasis Rev (1987) 5(3):185–204 10.1007/BF00046998 3549035

[38] Handa K Hakomori SI Carbohydrate to carbohydrate interaction in development process and cancer progression. Glycoconj J (2012) 29(8–9):627–37 10.1007/s10719-012-9380-7 22610315

[39] Baldus SE Engelmann K Hanisch FG MUC1 and the MUCs: a family of human mucins with impact in cancer biology. Crit Rev Clin Lab Sci (2004) 41(2):189–231 10.1080/10408360490452040 15270554

[40] Pinho S Marcos NT Ferreira B Carvalho AS Oliveira MJ Santos-Silva F Biological significance of cancer-associated sialyl-Tn antigen: modulation of malignant phenotype in gastric carcinoma cells. Cancer Lett (2007) 249(2):157–70 10.1016/j.canlet.2006.08.010 16965854

[41] Pinto R Carvalho AS Conze T Magalhaes A Picco G Burchell JM Identification of new cancer biomarkers based on aberrant mucin glycoforms by in situ proximity ligation. J Cell Mol Med (2012) 16(7):1474–84 10.1111/j.1582-4934.2011.01436.x 21883895 PMC3823216

[42] Mazal D Lo-Man R Bay S Pritsch O Deriaud E Ganneau C Monoclonal antibodies toward different Tn-amino acid backbones display distinct recognition patterns on human cancer cells. Implications for effective immuno-targeting of cancer. Cancer Immunol Immunother (2013) 62(6):1107–22 10.1007/s00262-013-1425-7 23604173 PMC11029704

[43] Kudo T Ikehara Y Togayachi A Morozumi K Watanabe M Nakamura M Up-regulation of a set of glycosyltransferase genes in human colorectal cancer. Lab Invest (1998) 78(7):797–811 9690558

[44] Tarp MA Clausen H Mucin-type O-glycosylation and its potential use in drug and vaccine development. Biochim Biophys Acta (2008) 1780(3):546–63 10.1016/j.bbagen.2007.09.010 17988798

[45] Wu YM Nowack DD Omenn GS Haab BB Mucin glycosylation is altered by pro-inflammatory signaling in pancreatic-cancer cells. J Proteome Res (2009) 8(4):1876–86 10.1021/pr8008379 19714813 PMC2893235

[46] Deguchi T Tanemura M Miyoshi E Nagano H Machida T Ohmura Y Increased immunogenicity of tumor-associated antigen, mucin 1, engineered to express alpha-gal epitopes: a novel approach to immunotherapy in pancreatic cancer. Cancer Res (2010) 70(13):5259–69 10.1158/0008-5472.CAN-09-4313 20530670

[47] Yu LG Andrews N Zhao Q McKean D Williams JF Connor LJ Galectin-3 interaction with Thomsen-Friedenreich disaccharide on cancer-associated MUC1 causes increased cancer cell endothelial adhesion. J Biol Chem (2007) 282(1):773–81 10.1074/jbc.M606862200 17090543

[48] Cascio S Farkas AM Hughey RP Finn OJ Altered glycosylation of MUC1 influences its association with CIN85: the role of this novel complex in cancer cell invasion and migration. Oncotarget (2013) 4(10):1686–97 24072600 10.18632/oncotarget.1265 PMC3858555

[49] Wang ZQ Bachvarova M Morin C Plante M Gregoire J Renaud MC Role of the polypeptide N-acetylgalactosaminyltransferase 3 in ovarian cancer progression: possible implications in abnormal mucin O-glycosylation. Oncotarget (2014) 5(2):544–60 24504219 10.18632/oncotarget.1652 PMC3964228

[50] Bennett EP Hassan H Mandel U Hollingsworth MA Akisawa N Ikematsu Y Cloning and characterization of a close homologue of human UDP-N-acetyl-alpha-D-galactosamine:polypeptide N-acetylgalactosaminyltransferase-T3, designated GalNAc-T6. Evidence for genetic but not functional redundancy. J Biol Chem (1999) 274(36):25362–70 10.1074/jbc.274.36.25362 10464263

[51] Gnemmi V Bouillez A Gaudelot K Hemon B Ringot B Pottier N MUC1 drives epithelial-mesenchymal transition in renal carcinoma through Wnt/beta-catenin pathway and interaction with SNAIL promoter. Cancer Lett (2013). 10.1016/j.canlet.2013.12.029 24384091

[52] Ponnusamy MP Seshacharyulu P Lakshmanan I Vaz AP Chugh S Batra SK Emerging role of mucins in epithelial to mesenchymal transition. Curr Cancer Drug Targets (2013) 13(9):945–56 10.2174/15680096113136660100 24168188 PMC3924542

[53] Rajabi H Alam M Takahashi H Kharbanda A Guha M Ahmad R MUC1-C oncoprotein activates the ZEB1/miR-200c regulatory loop and epithelial-mesenchymal transition. Oncogene (2013). 10.1038/onc.2013.114 23584475 PMC3783575

[54] Roy LD Sahraei M Subramani DB Besmer D Nath S Tinder TL MUC1 enhances invasiveness of pancreatic cancer cells by inducing epithelial to mesenchymal transition. Oncogene (2011) 30(12):1449–59 10.1038/onc.2010.526 21102519 PMC3063863

[55] Sikut R Nilsson O Baeckstrom D Hansson GC Colon adenoma and cancer cells aberrantly express the leukocyte-associated sialoglycoprotein CD43. Biochem Biophys Res Commun (1997) 238(2):612–6 10.1006/bbrc.1997.7334 9299561

[56] Fu Q Cash SE Andersen JJ Kennedy CR Oldenburg DG Zander VB CD43 in the nucleus and cytoplasm of lung cancer is a potential therapeutic target. Int J Cancer (2013) 132(8):1761–70 10.1002/ijc.27873 23015282

[57] Bendas G Borsig L Cancer cell adhesion and metastasis: selectins, integrins, and the inhibitory potential of heparins. Int J Cell Biol (2012) 2012:676731 10.1155/2012/676731 22505933 PMC3296185

[58] Gil-Bernabe AM Lucotti S Muschel RJ Coagulation and metastasis: what does the experimental literature tell us? Br J Haematol (2013) 162(4):433–41 10.1111/bjh.12381 23691951

[59] Wirtz D Konstantopoulos K Searson PC The physics of cancer: the role of physical interactions and mechanical forces in metastasis. Nat Rev Cancer (2011) 11(7):512–22 10.1038/nrc3080 21701513 PMC3262453

[60] Konstantopoulos K Thomas SN Cancer cells in transit: the vascular interactions of tumor cells. Annu Rev Biomed Eng (2009) 11:177–202 10.1146/annurev-bioeng-061008-124949 19413512

[61] Ugorski M Laskowska A Sialyl Lewis(a): a tumor-associated carbohydrate antigen involved in adhesion and metastatic potential of cancer cells. Acta Biochim Pol (2002) 49(2):303–11 12362971

[62] Sozzani P Arisio R Porpiglia M Benedetto C Is Sialyl Lewis x antigen expression a prognostic factor in patients with breast cancer? Int J Surg Pathol (2008) 16(4):365–74 10.1177/1066896908324668 18977761

[63] Sayat R Leber B Grubac V Wiltshire L Persad S O-GlcNAc-glycosylation of beta-catenin regulates its nuclear localization and transcriptional activity. Exp Cell Res (2008) 314(15):2774–87 10.1016/j.yexcr.2008.05.017 18586027

[64] Jin FZ Yu C Zhao DZ Wu MJ Yang Z A correlation between altered O-GlcNAcylation, migration and with changes in E-cadherin levels in ovarian cancer cells. Exp Cell Res (2013) 319(10):1482–90 10.1016/j.yexcr.2013.03.013 23524144

[65] Park SY Kim HS Kim NH Ji S Cha SY Kang JG Snail1 is stabilized by O-GlcNAc modification in hyperglycaemic condition. EMBO J (2010) 29(22):3787–96 10.1038/emboj.2010.254 20959806 PMC2989108

[66] Bei R Mizejewski GJ Alpha fetoprotein is more than a hepatocellular cancer biomarker: from spontaneous immune response in cancer patients to the development of an AFP-based cancer vaccine. Curr Mol Med (2011) 11(7):564–81 10.2174/156652411800615162 21707514

[67] Amri R Bordeianou LG Sylla P Berger DL Preoperative carcinoembryonic antigen as an outcome predictor in colon cancer. J Surg Oncol (2013) 108(1):14–8 10.1002/jso.23352 23681672

[68] Matsuura H Hakomori S The oncofetal domain of fibronectin defined by monoclonal antibody FDC-6: its presence in fibronectins from fetal and tumor tissues and its absence in those from normal adult tissues and plasma. Proc Natl Acad Sci U S A (1985) 82(19):6517–21 10.1073/pnas.82.19.6517 2995969 PMC390748

[69] Egeblad M Nakasone ES Werb Z Tumors as organs: complex tissues that interface with the entire organism. Dev Cell (2010) 18(6):884–901 10.1016/j.devcel.2010.05.012 20627072 PMC2905377

[70] Xie K Abbruzzese JL Developmental biology informs cancer: the emerging role of the hedgehog signaling pathway in upper gastrointestinal cancers. Cancer Cell (2003) 4(4):245–7 10.1016/S1535-6108(03)00246-0 14585350

[71] Radtke F Clevers H Self-renewal and cancer of the gut: two sides of a coin. Science (2005) 307(5717):1904–9 10.1126/science.1104815 15790842

[72] Bissell MJ Radisky D Putting tumours in context. Nat Rev Cancer (2001) 1(1):46–54 10.1038/35094059 11900251 PMC2975572

[73] Wiseman BS Werb Z Stromal effects on mammary gland development and breast cancer. Science (2002) 296(5570):1046–9 10.1126/science.1067431 12004111 PMC2788989

[74] Bissell MJ Labarge MA Context, tissue plasticity, and cancer: are tumor stem cells also regulated by the microenvironment? Cancer Cell (2005) 7(1):17–23 10.1016/j.ccr.2004.12.013 15652746 PMC2933216

[75] Bhowmick NA Neilson EG Moses HL Stromal fibroblasts in cancer initiation and progression. Nature (2004) 432(7015):332–7 10.1038/nature03096 15549095 PMC3050735

[76] Sternlicht MD Lochter A Sympson CJ Huey B Rougier JP Gray JW The stromal proteinase MMP3/stromelysin-1 promotes mammary carcinogenesis. Cell (1999) 98(2):137–46 10428026 10.1016/s0092-8674(00)81009-0 PMC2853255

[77] Paszek MJ Zahir N Johnson KR Lakins JN Rozenberg GI Gefen A Tensional homeostasis and the malignant phenotype. Cancer Cell (2005) 8(3):241–54 10.1016/j.ccr.2005.08.010 16169468

[78] Erler JT Weaver VM Three-dimensional context regulation of metastasis. Clin Exp Metastasis (2009) 26(1):35–49 10.1007/s10585-008-9209-8 18814043 PMC2648515

[79] Levental KR Yu H Kass L Lakins JN Egeblad M Erler JT Matrix crosslinking forces tumor progression by enhancing integrin signaling. Cell (2009) 139(5):891–906 10.1016/j.cell.2009.10.027 19931152 PMC2788004

[80] Matsuura H Takio K Titani K Greene T Levery SB Salyan MEK The oncofetal structure of human fibronectin defined by monoclonal antibody FDC-6: unique structural requirement for the antigenic specificity provided by a glycosylhexapeptide. J Biol Chem (1988) 263(7):3314–22 2449438

[81] Matsuura H Greene T Hakomori S An alpha-N-acetylgalactosaminylation at the threonine residue of a defined peptide sequence creates the oncofetal peptide epitope in human fibronectin. J Biol Chem (1989) 264(18):10472–6 2471705

[82] White ES Muro AF Fibronectin splice variants: understanding their multiple roles in health and disease using engineered mouse models. IUBMB Life (2011) 63(7):538–46 10.1002/iub.493 21698758

[83] Inufusa H Nakamura M Adachi T Nakatani Y Shindo K Yasutomi M Localization of oncofetal and normal fibronectin in colorectal cancer. Correlation with histologic grade, liver metastasis, and prognosis. Cancer (1995) 75(12):2802–8 10.1002/1097-0142(19950615)75:12<2802::AID-CNCR2820751204>3.0.CO;2-O 7773930

[84] Loridon-Rosa B Vielh P Matsuura H Clausen H Cuadrado C Burtin P Distribution of oncofetal fibronectin in human mammary tumors: immunofluorescence study on histological sections. Cancer Res (1990) 50:1608–12 2406016

[85] Mandel U Gaggero B Reibel J Therkildsen MH Dabelsteen E Clausen H Oncofetal fibronectins in oral carcinomas: correlation of two different types. APMIS (1994) 102(9):695–702 10.1111/j.1699-0463.1994.tb05222.x 7946273

[86] Freire-de-Lima L Gelfenbeyn K Ding Y Mandel U Clausen H Handa K Involvement of O-glycosylation defining oncofetal fibronectin in epithelial-mesenchymal transition process. Proc Natl Acad Sci U S A (2011) 108(43):17690–5 10.1073/pnas.1115191108 22006308 PMC3203762

[87] Ding Y Gelfenbeyn K Freire-de-Lima L Handa K Hakomori SI Induction of epithelial-mesenchymal transition with O-glycosylated oncofetal fibronectin. FEBS Lett (2012) 586(13):1813–20 10.1016/j.febslet.2012.05.020 22641031 PMC3377767

[88] Alisson-Silva F Freire-de-Lima L Donadio JL Lucena MC Penha L Sa-Diniz JN Increase of O-glycosylated oncofetal fibronectin in high glucose-induced epithelial-mesenchymal transition of cultured human epithelial cells. PLoS One (2013) 8(4):e60471 10.1371/journal.pone.0060471 23593224 PMC3625189

[89] Tsai JH Yang J Epithelial-mesenchymal plasticity in carcinoma metastasis. Genes Dev (2013) 27(20):2192–206 10.1101/gad.225334.113 24142872 PMC3814640

[90] Karamitopoulou E Role of epithelial-mesenchymal transition in pancreatic ductal adenocarcinoma: is tumor budding the missing link? Front Oncol (2013) 3:221 10.3389/fonc.2013.00221 24062980 PMC3774985

[91] Catalano V Turdo A Di Franco S Dieli F Todaro M Stassi G Tumor and its microenvironment: a synergistic interplay. Semin Cancer Biol (2013) 23(6 Pt B):522–32 10.1016/j.semcancer.2013.08.007 24012661

[92] Creighton CJ Gibbons DL Kurie JM The role of epithelial-mesenchymal transition programming in invasion and metastasis: a clinical perspective. Cancer Manag Res (2013) 5:187–95 10.2147/CMAR.S35171 23986650 PMC3754282

[93] Zeisberg M Neilson EG Biomarkers for epithelial-mesenchymal transitions. J Clin Invest (2009) 119(6):1429–37 10.1172/JCI36183 19487819 PMC2689132

[94] Thiery JP Acloque H Huang RJY Nieto MA Epithelial-mesenchymal transitions in development and disease. Cell (2009) 139(5):871–90 10.1016/j.cell.2009.11.007 19945376

[95] Kalluri R Weinberg RA The basics of epithelial-mesenchymal transition. J Clin Invest (2009) 119(6):1420–8 10.1172/JCI39104 19487818 PMC2689101

[96] Klymkowsky MW Savagner P Epithelial-mesenchymal transition: a cancer researcher’s conceptual friend and foe. Am J Pathol (2009) 174(5):1588–93 10.2353/ajpath.2009.080545 19342369 PMC2671246

[97] Guan F Handa K Hakomori S Specific glycosphingolipids mediate epithelial-to-mesenchymal transition of human and mouse epithelial cell lines. Proc Natl Acad Sci U S A (2009) 106(18):7461–6 10.1073/pnas.0902368106 19380734 PMC2678670

[98] Guan F Schaffer L Handa K Hakomori S Functional role of gangliotetraosylceramide in epithelial-to-mesenchymal transition process induced by hypoxia and by TGF-beta. FASEB J (2010) 24(12):4889–903 10.1096/fj.10-162107 20720159 PMC2992377

[99] Liang YJ Ding Y Levery SB Lobaton M Handa K Hakomori SI Differential expression profiles of glycosphingolipids in human breast cancer stem cells vs. cancer non-stem cells. Proc Natl Acad Sci U S A (2013) 110(13):4968–73 10.1073/pnas.1302825110 23479608 PMC3612608

[100] Maupin KA Sinha A Eugster E Miller J Ross J Paulino V Glycogene expression alterations associated with pancreatic cancer epithelial-mesenchymal transition in complementary model systems. PLoS One (2010) 5(9):e13002 10.1371/journal.pone.0013002 20885998 PMC2946336

[101] Pinho SS Oliveira P Cabral J Carvalho S Huntsman D Gartner F Loss and recovery of Mgat3 and GnT-III mediated E-cadherin N-glycosylation is a mechanism involved in epithelial-mesenchymal-epithelial transitions. PLoS One (2012) 7(3):e33191 10.1371/journal.pone.0033191 22427986 PMC3302839

[102] Lin H Wang D Wu T Dong C Shen N Sun Y Blocking core fucosylation of TGF-beta1 receptors downregulates their functions and attenuates the epithelial-mesenchymal transition of renal tubular cells. Am J Physiol Renal Physiol (2011) 300(4):F1017–25 10.1152/ajprenal.00426.2010 21228108

[103] Xu Q Isaji T Lu Y Gu W Kondo M Fukuda T Roles of N-acetylglucosaminyltransferase III in epithelial-to-mesenchymal transition induced by transforming growth factor beta1 (TGF-beta1) in epithelial cell lines. J Biol Chem (2012) 287(20):16563–74 10.1074/jbc.M111.262154 22451656 PMC3351319

[104] Richter P Junker K Franz M Berndt A Geyer C Gajda M IIICS de novo glycosylated fibronectin as a marker for invasiveness in urothelial carcinoma of the urinary bladder (UBC). J Cancer Res Clin Oncol (2008) 134(10):1059–65 10.1007/s00432-008-0390-6 18386055 PMC12161765

[105] Giovannucci E Harlan DM Archer MC Bergenstal RM Gapstur SM Habel LA Diabetes and cancer: a consensus report. Diabetes Care (2010) 33(7):1674–85 10.2337/dc10-0666 20587728 PMC2890380

[106] Shikata K Ninomiya T Kiyohara Y Diabetes mellitus and cancer risk: review of the epidemiological evidence. Cancer Sci (2010) 104(1):9–14 10.1111/cas.12043 23066889 PMC7657146

[107] Peairs KS Barone BB Snyder CF Yeh HC Stein KB Derr RL Diabetes mellitus and breast cancer outcomes: a systematic review and meta-analysis. J Clin Oncol (2010) 29(1):40–6 10.1200/JCO.2009.27.3011 21115865 PMC3055858

[108] Xue F Michels KB Diabetes, metabolic syndrome, and breast cancer: a review of the current evidence. Am J Clin Nutr (2007) 86(3):s823–35 18265476 10.1093/ajcn/86.3.823S

[109] Currie CJ Poole CD Jenkins-Jones S Gale EA Johnson JA Morgan CL Mortality after incident cancer in people with and without type 2 diabetes: impact of metformin on survival. Diabetes Care (2012) 35(2):299–304 10.2337/dc11-1313 22266734 PMC3263862

[110] Jiralerspong S Kim ES Dong W Feng L Hortobagyi GN Giordano SH Obesity, diabetes, and survival outcomes in a large cohort of early-stage breast cancer patients. Ann Oncol (2013) 24(10):2506–14 10.1093/annonc/mdt224 23793035 PMC3784334

[111] Singh S Singh PP Singh AG Murad MH McWilliams RR Chari ST Anti-diabetic medications and risk of pancreatic cancer in patients with diabetes mellitus: a systematic review and meta-analysis. Am J Gastroenterol (2013) 108(4):510–9 10.1038/ajg.2013.7 23399556

[112] Onitilo AA Engel JM Glurich I Stankowski RV Williams GM Doi SA Diabetes and cancer II: role of diabetes medications and influence of shared risk factors. Cancer Causes Control (2012) 23(7):991–1008 10.1007/s10552-012-9971-4 22527174 PMC4138811

[113] Kim YH Ryu JM Lee YJ Han HJ Fibronectin synthesis by high glucose level mediated proliferation of mouse embryonic stem cells: involvement of ANG II and TGF-beta1. J Cell Physiol (2010) 223(2):397–407 10.1002/jcp.22048 20112290

[114] Lin S Sahai A Chugh SS Pan X Wallner EI Danesh FR High glucose stimulates synthesis of fibronectin via a novel protein kinase C, Rap1b, and B-Raf signaling pathway. J Biol Chem (2002) 277(44):41725–35 10.1074/jbc.M203957200 12196513

[115] Kolm-Litty V Sauer U Nerlich A Lehmann R Schleicher ED High glucose-induced transforming growth factor beta1 production is mediated by the hexosamine pathway in porcine glomerular mesangial cells. J Clin Invest (1998) 101(1):160–9 10.1172/JCI119875 9421478 PMC508552

[116] Rocco MV Chen Y Goldfarb S Ziyadeh FN Elevated glucose stimulates TGF-beta gene expression and bioactivity in proximal tubule. Kidney Int (1992) 41(1):107–14 10.1038/ki.1992.14 1593845

[117] Smoak IW Hyperglycemia-induced TGFbeta and fibronectin expression in embryonic mouse heart. Dev Dyn (2004) 231(1):179–89 10.1002/dvdy.20123 15305298

[118] Altschul SF Madden TL Schaffer AA Zhang J Zhang Z Miller W Gapped BLAST and PSI-BLAST: a new generation of protein database search programs. Nucleic Acids Res (1997) 25(17):3389–402 10.1093/nar/25.17.3389 9254694 PMC146917

[119] Shaw JL Diamandis EP Distribution of 15 human kallikreins in tissues and biological fluids. Clin Chem (2007) 53(8):1423–32 10.1373/clinchem.2007.088104 17573418

[120] Kryza T Achard C Parent C Marchand-Adam S Guillon-Munos A Iochmann S Angiogenesis stimulated by human kallikrein-related peptidase 12 acting via a platelet-derived growth factor B-dependent paracrine pathway. FASEB J (2014) 28(2):740–51 10.1096/fj.13-237503 24225148

[121] Lu Z Cui M Zhao H Wang T Shen Y Dong Q Tissue kallikrein mediates neurite outgrowth through epidermal growth factor receptor and flotillin-2 pathway in vitro. Cell Signal (2014) 26(2):220–32 10.1016/j.cellsig.2013.10.010 24211626

[122] Borgono CA Diamandis EP The emerging roles of human tissue kallikreins in cancer. Nat Rev Cancer (2004) 4(11):876–90 10.1038/nrc1474 15516960

[123] Batra J O’Mara T Patnala R Lose F Clements JA Genetic polymorphisms in the human tissue kallikrein (KLK) locus and their implication in various malignant and non-malignant diseases. Biol Chem (2012) 393(12):1365–90 10.1515/hsz-2012-0211 23667899

[124] Kuzmanov U Jiang N Smith CR Soosaipillai A Diamandis EP Differential N-glycosylation of kallikrein 6 derived from ovarian cancer cells or the central nervous system. Mol Cell Proteomics (2009) 8(4):791–8 10.1074/mcp.M800516-MCP200 19088065 PMC2667357

[125] Goletz S Hanisch FG Karsten U Novel alphaGalNAc containing glycans on cytokeratins are recognized invitro by galectins with type II carbohydrate recognition domains. J Cell Sci (1997) 110(Pt 14):1585–96 9247192 10.1242/jcs.110.14.1585

[126] Chou CF Smith AJ Omary MB Characterization and dynamics of O-linked glycosylation of human cytokeratin 8 and 18. J Biol Chem (1992) 267(6):3901–6 1371281

[127] Hatsell S Medina L Merola J Haltiwanger R Cowin P Plakoglobin is O-glycosylated close to the N-terminal destruction box. J Biol Chem (2003) 278(39):37745–52 10.1074/jbc.M301346200 12847106

[128] Hu P Berkowitz P Madden VJ Rubenstein DS Stabilization of plakoglobin and enhanced keratinocyte cell-cell adhesion by intracellular O-glycosylation. J Biol Chem (2006) 281(18):12786–91 10.1074/jbc.M511702200 16510446

[129] Lommel M Winterhalter PR Willer T Dahlhoff M Schneider MR Bartels MF Protein O-mannosylation is crucial for E-cadherin-mediated cell adhesion. Proc Natl Acad Sci U S A (2013) 110(52):21024–9 10.1073/pnas.1316753110 24297939 PMC3876218

[130] Ha JR Hao L Venkateswaran G Huang YH Garcia E Persad S β-Catenin is O-GlcNAc glycosylated at serine 23: implications for beta-catenin’s subcellular localization and transactivator function. Exp Cell Res (2014) 321(2):153–66 10.1016/j.yexcr.2013.11.021 24342833

